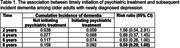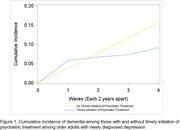# Timely Initiation of Psychiatric Treatment and Subsequent Incident Dementia in Older Adults with Newly Diagnosed Depression: The Health and Retirement Study 2012‐2020

**DOI:** 10.1002/alz70860_097830

**Published:** 2025-12-23

**Authors:** Jingkai Wei, Youngran Kim, Yanan Zhang, Victoria Tang, Matthew Lohman, Anwar T Merchant, Casey Crump

**Affiliations:** ^1^ University of Texas Health Science Center at Houston, Houston, TX, USA; ^2^ University of South Carolina, Columbia, SC, USA

## Abstract

**Background:**

Late‐life depression is a well‐established risk factor for dementia. While timely treatment of late‐life depression is critical for improving mental health outcomes, evidence for whether it reduces dementia risk is limited. This study emulated a target trial to estimate the association between timely initiation of psychiatric treatment and risk of dementia among older adults with newly diagnosed depression.

**Method:**

Participants aged ≥60 years from the Health and Retirement Study from 2012 to 2018 with newly diagnosed depression (within 2 years) but no history of dementia or previous psychiatric treatment were included and followed up through 2020. Newly diagnosed late‐life depression and incident dementia were self‐reported through questionnaires. Timely initiation of psychiatric treatment was defined as self‐reported initiation at the same wave as the depression diagnosis. Logistic regression was used to estimate the cumulative incidence of dementia in both groups (i.e., timely initiation vs. no timely initiation of psychiatric treatment) using stabilized inverse probability of treatment and censoring weights to emulate random treatment assignment and control for confounding. Risk ratios (RRs) were calculated based on estimated cumulative incidence of dementia, and 95% confidence intervals (CIs) were calculated with 200 sets of bootstrapping.

**Result:**

Among 1,129 participants with newly diagnosed depression from 2012 to 2018 (mean age: 72 years; 61% women; 65% non‐Hispanic White), 216 (19.1%) reported timely initiation of psychiatric treatment. The cumulative incidence of dementia over 8 years was lower among those with timely initiation of psychiatric treatment (9.2%) compared to those with no treatment (15.8%), with an RR = 0.58 (95% CI: 0.29–1.00). Risk of dementia steadily decreased over 8 years following timely initiation of psychiatric treatment for newly diagnosed depression (Figure 1, Table 1).

**Conclusion:**

Timely initiation of psychiatric treatment was associated with a lower risk of subsequent dementia among older adults with newly diagnosed depression, further highlighting the importance of increased screening and early psychiatric intervention in managing late‐life depression.